# Exploring Twitter to Analyze the Public’s Reaction Patterns to Recently Reported Homicides in London

**DOI:** 10.1371/journal.pone.0121848

**Published:** 2015-03-26

**Authors:** Ourania Kounadi, Thomas J. Lampoltshammer, Elizabeth Groff, Izabela Sitko, Michael Leitner

**Affiliations:** 1 Doctoral College GIScience, Department of Geoinformatics-Z_GIS, University of Salzburg, Salzburg, Austria; 2 School of Information Technology and Systems Management, Salzburg University of Applied Sciences, Salzburg, Austria; 3 Department of Criminal Justice, Temple University, Philadelphia, Pennsylvania, United States of America; 4 Department of Geography and Anthropology, Louisiana State University, Baton Rouge, Louisiana, United States of America; UNMdP-CONICET, ARGENTINA

## Abstract

Crime is an ubiquitous part of society. The way people express their concerns about crimes has been of particular interest to the scientific community. Over time, the numbers and kinds of available communication channels have increased. Today, social media services, such Twitter, present a convenient way to express opinions and concerns about crimes. The main objective of this study is to explore people’s perception of homicides, specifically, how the characteristics and proximity of the event affect the public’s concern about it. The analysis explores Twitter messages that refer to homicides that occurred in London in 2012. In particular, the dependence of tweeting propensity on the proximity, in space and time, of a crime incident and of people being concerned about that particular incident are examined. Furthermore, the crime characteristics of the homicides are analysed using logistic regression analysis. The results show that the proximity of the Twitter users’ estimated home locations to the homicides’ locations impacts on whether the associated crime news is spread or not and how quickly. More than half of the homicide related tweets are sent within the first week and the majority of them are sent within a month of the incident’s occurrence. Certain crime characteristics, including the presence of a knife, a young victim, a British victim, or a homicide committed by a gang are predictors of the crime-tweets posting frequency.

## Introduction

This study contributes to the existing literature of crime perception. The main objective is to explore citizen’s perception of homicides. In particular, how the characteristics and proximity of the event affect the public’s concern about it as reflected by the timing and content of tweets. Our research fits into the topics of fear of crime, which is addressed as a psychological reaction [1–3] and concern about crime, which is considered a social problem [4–5].

Regarding the spatio-temporal aspects of crime perception, we want to examine if there is any dependence on the proximity in space or time of a crime incident, and the people who are concerned about that particular incident. The study of Lewis and Maxfield [6] surveyed residents in four Chicago neighborhoods and revealed that the fear of crime is a problem independent of the actual crime rates. In addition, Furstenberg [5] revealed that people living in low crime areas are more concerned about crime than people living in high crime areas. Further studies examined how landscape patterns affect the fear of crime and crime prevalence estimates. The results of Wang and Taylor [7] showed that safety concerns varied as respondents walk down two different urban alleys that varied in terms of their lengths, land use patterns, and site features. Controlling for position in the alley, two elements were linked with the amount of fear respondents experienced. These were the expected direction to refuge for attackers, a composite element of the proximity of escape routes and how well one can see what is coming. McCord et al. [8] focused on variations in land use and confirmed a positive association between the “crime-generator index” or the “crime-attractor index” and of perceived incivilities and crime. Similarly, Williams [9] surveyed residents of a small geographical area in London regarding concern about crime types. The results were mapped so as to reveal variations in the level of fear of crime in space. The author and the residents interpreted these variations based on environmental and landscape characteristics (e.g. “the streets are dirty”). Furthermore, to the best of our knowledge there is only one study that addresses the relationship between the temporal behavior of people concerned about a crime, and the temporal attributes of the crime itself. Heverin and Zach [10] examined Twitter messages that referred to the shooting of four police officers in Washington State, USA in November 2009. Relevant tweets were retrieved for the next two and a half days after the event, for five time periods of twelve hours each. Then, the tweets were categorized by types (e.g. emotion or opinion) and the percentages of each type per time period were analyzed. The authors proposed that text mining of microblogging is a promising method to disseminate crisis-related information. With respect to crime characteristics, we want to investigate if certain characteristics are associated with the public’s higher or lower concerns. Empirical studies showed that characteristics of crimes affect people’s reactions, feelings, and opinions about them. For instance, official rates of violent crimes [11] and direct victimization [5] are positively related to fear of crime. Other explanatory variables of crime perception that have been examined in the literature are: a) locality, sensationalism, and randomness of crime [12–14], b) types of crime visualization [15], and c) whether justice is restored or not [16].

To address our research objective, we use two data sources. The first one is crime reports from online newspapers. Newspapers were chosen since previous studies in several regions worldwide [17–19], as well as in the UK, which is the study area of this research [14], [20], [21], suggest that media affects peoples’ perception. More important, both in the UK [21] and elsewhere [15] media, and in particular newspapers, are the primary source of crime-related information. The second data source is the Twitter social network and microblogging service that is used to explore public’s concerns about crime reports. Recent publications applied data mining techniques to social media content to examine perceptions and other behavioral patterns of individuals over several topics of interest. Amongst these topics, the use of social media regarding health related research [22–24], risk management and natural disasters [25–27] have gained increasing interest in the current literature. Also, social media platforms have been used as a tool to sense collective human behavior and uncover mobility patterns at different geographical scales [28], [29]. Other studies focused on developing topic detection techniques [30], [31], which identify clustering of words that frequently appear together in large volumes of unstructured text. Others explored the geo-coded portion of social media to show its novel potential for geographical analysis and visualization [32–35]. Furthermore, context from social media has been used to mine information for crime analysis and investigation. White and Roth [36] presented the application “TwitterHitter” that creates geovisual analytics to harvest topics of interest. The application was demonstrated for the purposes of crime analysis and the authors provided examples of how it may be used for criminal investigative analysis, intelligence analysis, tactical analysis, strategic analysis, and administrative analysis. Sun and Ng [37] developed the “Comment Arrival Model”, which reveals the social media messages’ popularity. They analyzed the "drug abuse" topic and suggested that the model can be used for crime detection. Concerning crime prediction, Wang et al. [38] used Twitter data combined with natural language techniques and liner modeling as an alternative approach to traditional methods for crime prediction. Said et al. [39] extracted and analyzed social networking data files using different types of smart phones and proposed to forensic analysts and security specialists the great potential of social media mining for digital crime investigation. Last, with regards to text mining in crime intelligence analysis, Helbich et al. [40] proposed an innovative approach using self-organizing maps and point pattern analysis. The objective of their research was to explore information extracted from people’s statements regarding the last victim of a homicide series in Jennings, Louisiana in the USA.

Our motivation to explore crime perception is based on literature findings that reveal variations of perception in several dimensions such as spatial, temporal, and crime characteristics. Our approach is to combine data sources (i.e. media reports and social media text) as an alternative to traditional sources (surveys or census data). Finally, our research utilizes methods that were developed by Lampoltshammer et al. [41]. The authors analyzed variations of crime perception by crime type, demographic, and spatial characteristics. In our study we adopt the data-processing methods “*Links Correspondence Method*” and “*Home Estimation Method*” and build upon the limitations and suggestions mentioned by the aforementioned authors. Firstly, the validation of the previous study’s results was limited due to the small available sample size. Secondly, empirical results indicate that violent crime has a stronger effect on people’s perceptions than other crime types [13], [41]. Consequently, we analyze and validate an “as complete as possible” dataset that contains homicides reports. Also, we develop an extended version of the analytical methodology. We believe that the additional analytical parts of this study account for the most important aspects influencing the public’s concern about crime. The first aspect is the temporal variations of concerns and the second aspect is the spatial variations of concerns. Here we want to investigate the spatio-temporal relationships between homicides-related tweets and homicide incidents. The third aspect is the variations of concerns due to variations in crime characteristics. In particular, we want to examine whether the characteristics of homicides can explain the public’s reaction to crime news on Twitter. Details about the study’s data sources and primary datasets are described in **Section 2**. Then we present, in separate sections (**3–5**), the additional databases, subsets, methods, and analyses that were used to address each of the three aspects. Last, **Section 6** discusses the study’s findings.

## Data

This section presents the acquisition and processing steps of the data sources to obtain the study’s primary datasets. As the basis for our study we stored all homicide cases (in total 98 incidents) from “www.murdermap.co.uk” from the year 2012 [42] in our *Homicide-Dataset*. This website is dedicated to mapping every single homicide in London, starting from the crime itself up to the conviction of the perpetrator. The operators of the website acquire the information directly from the police, reports in media, and court reports. All information shared via the website is freely available. The information contains details about the victim (name, gender, date of crime, nationality, and the homicide’s weapon), as well as about the perpetrator (name, gender, and nationality). In addition, the status of the crime (unsolved/solved), the location of the incident, and a category of the general type of crime are provided.

We searched up to ten web links that pointed to respective media reports. We selected, and then verified, the links based on a maximum of the first 10 URLs (uniform resource locator) that were found with Google search using keywords from the incidents. Keywords included but were not limited to combinations such as: perpetrator’s name and victim’s name or weapon and location and victim’s gender and date. Several combinations were applied to obtain as many URLs as possible for each incident. The majority of the URLs were retrieved from UK-based online newspapers and few URLs from blogs and other websites located in the UK or in other countries. Even though all incidents were publicly available and reported on the internet, we retrieved different numbers and types of links for each homicide. For instance, we found many links for some homicides while for others only a few. Some were covered by worldwide and well-known newspapers, while others by local newspapers or small blogs. The number of links varied significantly also for the incidents that were covered by well-known newspapers. So far evidence shows that homicides vary in popularity, which justifies the initial objective to compare them.

In total 781 web crime links were collected, which served as a look-up table to connect Twitter messages with the homicide cases. In the next step, we searched for Twitter messages that contain those news links and therefore spread the information within the social network community. Twitter messages were collected from the social media reseller ‘TOPSY’ [43]. TOPSY is a social search analytics company and also a certified Twitter partner. Its commercial application (*Topsy Pro Analytics*) allows users to collect relevant twitter messages with keyword-based queries. In our case the keywords were the 781 web crime links. The data as such were collected on February 23rd 2014 from the TOPSY service. As this service provides access to all tweets back from 2006 up to now, completeness can be assumed for our data set. The results were then exported from TOPSY as a CSV file for further processing. The exported Twitter messages dataset was cleaned in terms of removing tweets from professional organizations, newspapers, and commercial sources. This formed our *HomicideTwitter-Dataset*. The S1 Dataset includes the 98 crime incidents (Homicide-Dataset) and the 781 web crime links (Murder links) that were used to extract the HomicideTwitter-Dataset. The *HomicideTwitter-Dataset* contains 216 links from the look-up table, 70 homicides incidents from the *Homicide-Dataset*, and in total 3,272 tweets. The tweets were sent by 1,939 users posting on an average 1.7 tweets (S.D = 2.4) from the 2^nd^ of January 2012 until the 4^th^ of February 2014.

## Temporal Analysis

**Fig 1:**
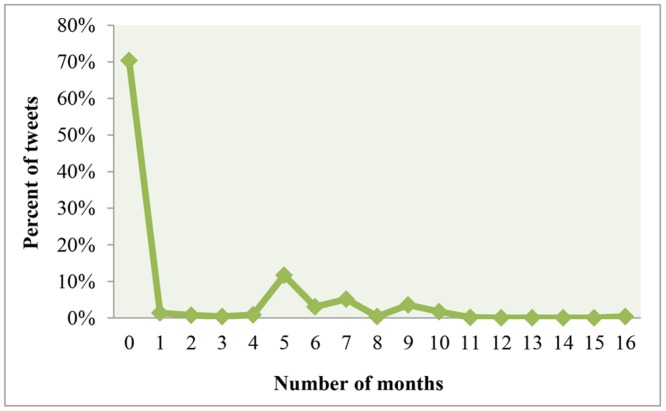
Distribution of Twitter responses to homicide incidents in months. 0 shows the percent of tweets that were sent within 30 days of the homicide’s occurrence, 1 shows the percent of tweets that were sent within 31–60 days, etc.

**Fig 2:**
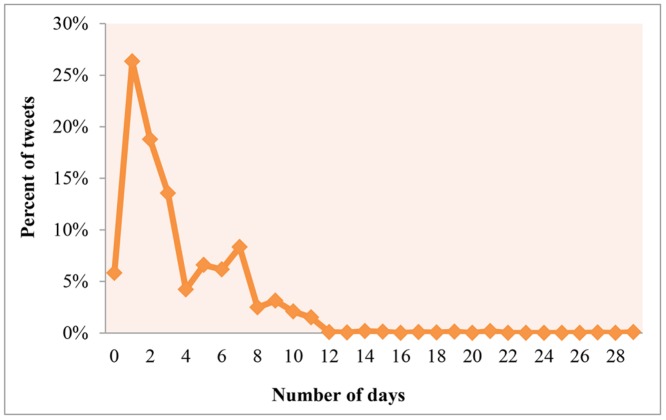
Distribution of Twitter response to homicide incidents in days. The distribution refers to tweets that were sent within the first month of the incident. 0 depicts the percent of tweets that were sent the same day of the homicide’s occurrence, 1 indicates the percent of tweets that were sent the next day, etc.

In this section we examined the public’s temporal reaction on Twitter regarding homicide incidents. The analysis aims to detect and discuss temporal peaks. Furthermore, the results may indicate a time frame for which Twitter can be mined in future research to assist homicide crime investigations. Firstly, we analyzed the length of the time period in days and months between the actual homicide incidents and the public’s reaction to them on Twitter (Figs. 1 and 2).

As it can be seen from Fig. 1, 70% of all tweets were posted within one month of the occurrence of the incidents. In addition, the figure reveals a small peak occurring after five months (151–180 days) with about 12% of tweets. This reflects the reaction of people to additional breaking news about the homicides such as confessions and convictions. In our data set, a mother pleaded guilty of killing her children. A similar case occurred in the smaller peak after seven months (211–240 days), when a brother confessed the murder of his sister. Changing the temporal scale, Fig. 2 zooms in on the daily distribution of the tweets that were posted within one month (30 days) of the homicide occurrences. It can be seen that about 81% of the tweets within the first month were sent within the first week of the homicide occurrence. The highest peak appears on the first day after the incident with about 26%. Altogether, this indicates that Twitter users disseminate information about homicides close to the date when the actual incident occurred.

**Fig 3:**
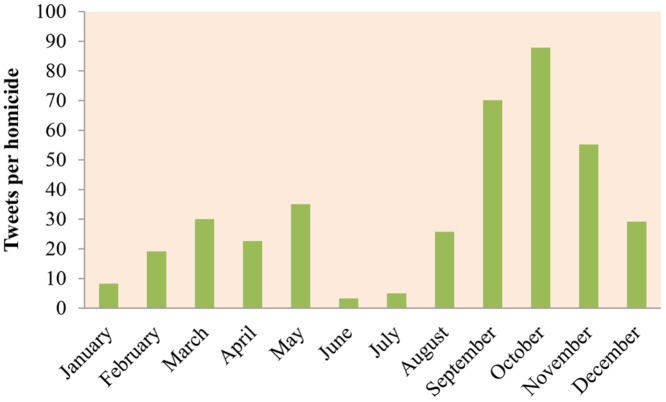
Homicide tweets ratio by month in 2012.

In the next step we calculated the percent of tweets that occurred in the same month as the homicides that they refer to (e.g. homicide occurred in June and the tweet that refers to the homicide was posted in June). This was 65% of the tweets, which implies that the homicides are being discussed on Twitter mainly at the month of their occurrence. Then, the tweets per homicide ratios were calculated over a period of twelve months in 2012 (Fig. 3). These ratios are calculated as the number of homicide-tweets per month divided by the number of homicides that occurred in the same month. Equal interest or concern about homicides would yield similar ratios per month. However, in contrast, a great variation in the ratios across the twelve months is observed. In June we have the lowest ratio with 3.3 tweets per homicide (46 tweets and 14 homicides) while in October the ratio is 87.9 tweets per homicide (703 tweets and 8 homicides). Also, during the autumn a higher response is observed compared to other seasons. This is a first indication that certain homicides receive more attention than others.

## Spatial Analysis

**Fig 4:**
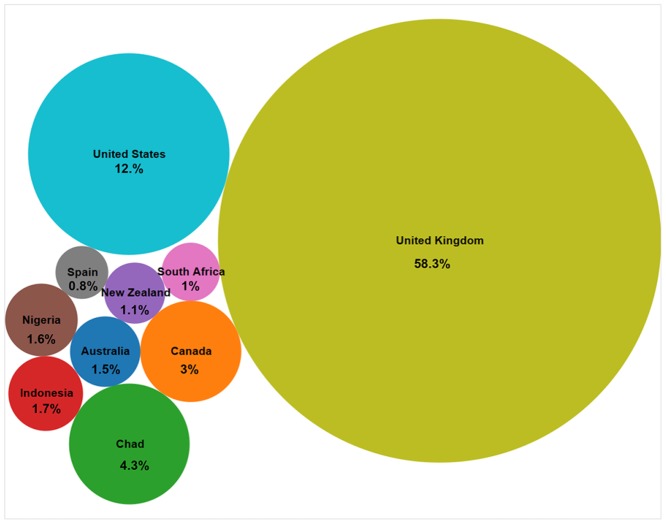
Share of tweets that refer to London’s homicide news among different countries.

This section presents an analysis of the relationship between the users’ locations and the locations of the homicides. For a general overview of the worldwide response to local crime news, we relied on the location information extracted from the users’ profiles in Twitter. After cleaning the data to remove empty and irrelevant entries (e.g. jokes) from the location field on the users’ profiles, 1,088 tweets were used for the global analysis (ca. 33% of the original *HomicideTwitter-Dataset*). The locations referred to different geographical scales, from a national to a neighborhood level. In order to achieve an equal granularity we extracted the name of the countries using the free Web mapping service “Nominatim” [44]. In total, homicides that occurred in London in 2012 were commented by messages coming from 74 countries. Nevertheless, only 10 countries accounted for more than 85% of all tweets (Fig. 4). The results clearly show that even though London is one of the world’s largest cities and a metropolitan area, the influence of the information about the crimes strongly depends on the locality, the spoken language, and the particular attributes of the crime incident. As it was anticipated, the majority of tweets were sent from the UK. Among other countries with a high frequency of crime tweets are countries where English is the first official language, or one of the official languages (USA, South Africa, and Nigeria). Furthermore, after comparing information in the *Homicide-Dataset* with some of the countries with high frequency (e.g. Australia, Canada, and New Zealand), we observed that these countries are related to the nationality of the victim or the perpetrator of the homicides. To a minor extent, observed frequencies may be affected by the unequal adoption of Twitter among different nations, with the generally higher popularity of this medium in wealthier countries, especially in the UK and the USA [45], [29].

Furthermore, a local spatial analysis was performed to examine whether people living closer to locations of crime incidents exhibit higher tendency to discuss them on Twitter. In order to estimate home locations of users who created messages gathered from the *HomicideTwitter-Dataset* we examined their geo-located posts from an additional database, the *geo-located Twitter database*. The *geo-located Twitter database* is a subset of the Twitter database that consists of tweets with location information at the level of geographic coordinates. It includes all geo-located messages from the UK in 2012 (in total 68,579,232), collected directly from the Twitter Streaming API [46]. This snapshot from the entire Twitter database was cleaned from errors and artificial tweeting noise, similarly as described by Hawelka et al. [29]. Since the analysis was restricted to London, all geo-located tweets outside the city were excluded. Also, in order to obtain a realistic subset of the users’ potential home locations we excluded daytime hours (07:00 to 17:59), since some users may frequently post from their work locations. Finally, we excluded all users who had less than ten geo-located messages. In total, the locations of 13,830 geo-located tweets formed the *night activity spaces* of 94 users who posted about 124 tweets related to the 70 homicides. The number of geo-located tweets per user ranges from 10 to 886.

This set of 13,830 locations was further analyzed to estimate the home location of each user from the *HomicideTwitter-Dataset*. The estimation method consisted of three steps and was repeatedly applied to each user. Firstly, a spatial clustering was performed using the “Nearest Neighbor Hierarchical Clustering” method [47]. The following parameters were used with this method: one standard deviational ellipses to outline the clusters, a minimum of 10 points per cluster, only first-order clusters, and a search radius based on the random nearest neighbor distance. Secondly, locations contained in the highest frequency cluster were selected. Finally, the center of minimum distance of these locations was calculated. This is the point that possesses the smallest accumulated distance to all other points in the cluster and from here on it is called “central feature”. In few cases where no significant clusters were found, the central feature was directly calculated. We assume that central features are likely to be the user’s home locations, since tweets are sent during evening times. This is an approximation based on reasoning of common human habits and may not be the case for all users. If some of these places are not the “true” home locations, they are still the most frequently visited places and locations that are of high interest to users.

**Fig 5:**
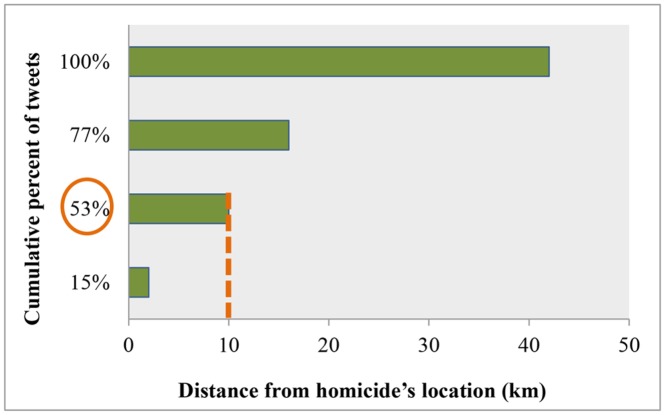
The cumulative percent of crime tweets at different distance intervals. Distance is defined as the distance from users’ estimated home locations to the crime incidents’ locations.

**Fig 6:**
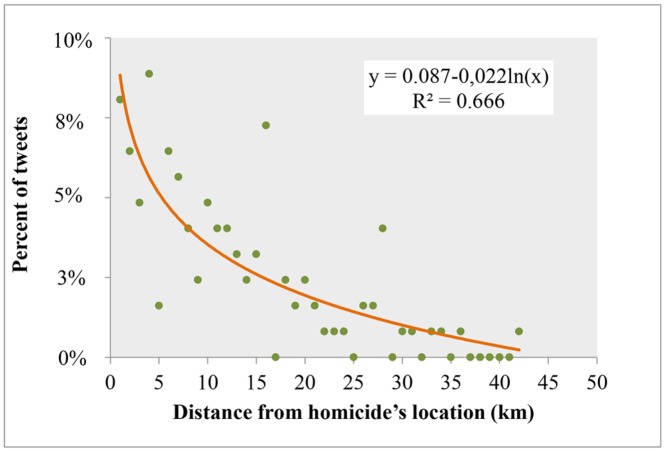
The distance decay fitted model between the frequency of the tweets and the distance from crime incidents. Distance is defined as the distance from users’ estimated home locations to the crime incidents’ locations.

Then, the Euclidean distances between the users’ estimated home locations and the locations of crimes were calculated and further explored. The results of the distance analysis between the users’ locations and the homicides locations confirm that the proximity to the crime incident affects the user’s decision on posting about it. More specifically, authors of 53% of crime-tweets live within a 10 km buffer zone around the crime’s location, while authors of the remaining tweets live within a buffer zone of greater than 10 km to 42 km (Fig. 5). The cumulative distribution shows that tweets are more associated with shorter distances to homicide locations, rather than with longer ones. Furthermore, we plotted the frequency of the tweets against the distance from users’ estimated home locations to the homicide locations (Fig. 6). The graph clearly shows a decreasing trend, which also indicates spatial dependency. In other words, the closer a user resides to a crime location the more likely it is that he or she will spread homicide news via Twitter. Lastly, a distance decay model was fitted for this type of relationship at a distance aggregation of 1 km (Fig. 6). Among several functions, the one that best fits the data is the logarithmic function (R^2^ = 0.666, F = 79.685, b = -0.22, constant = 0.087, at p < 0.001).

## Frequency Analysis

The results of the temporal analysis showed that the monthly distribution of crime tweets does not follow the monthly distribution of crimes. This indicates that even though all crimes are of the same type, namely homicides, they do not have the same impact on Twitter users. A high volume of tweets for a particular crime could imply high popularity of this topic, great interest, and consequently higher concern about this incident. To analyze the distribution of tweets and understand what makes some incidents receive more attention than others we use an ordinal logistic regression (OLR) analysis.

The dependent variable of the analysis is the frequency of the tweets of each crime incident. Ordinal logistic regression requires the dependent variable to be of ordinal type. However, it can be used when a categorical variable has been derived from a measured continuous variable [48]. According to Agresti [49] logistic regression is the most important model for categorical response data and it has been used in a variety of applications such as biomedical studies, as well as social science research and marketing. To use ordinal logistic regression we categorized the number of tweets per crime incident using the “natural breaks” classification, where the lowest values after peaks in a distribution are used to define classes’ limits. The “natural breaks” model was proposed by Jenks & Caspall [50] and follows a similar concept to the approach of Fisher [51], which minimizes the variance within the classes to create homogeneous groups. We selected a number of classes that balances detailed information with sufficient observations per class. This resulted in the following five frequency classes: homicides with a) 0–8 tweets, b) 9–26 tweets, c) 27–55 tweets, d) 56–114 tweets, and e) 115–420 tweets.

As for the independent variables, we used information about crimes that was obtained from the source website [42]. The information was organized in nine variables: 1) victim’s gender, 2) victim’s age, 3) victim’s nationality, 4) perpetrator’s gender, 5) perpetrator’s nationality, 6) number of arrested perpetrators, 7) weapon, 8) category of homicide, and 9) status of conviction. In particular, the first three variables describe demographic characteristics of victims, the next two describe demographic characteristics of perpetrators, and the last four describe specific characteristics of the crime itself. The weapon variable defines the type of the weapon that was used (e.g. none or knife), the “category of homicide” defines the type of the homicide (e.g. drug related or domestic), and the last variable “status of homicide” clarifies whether the crime incident is a solved or an unsolved case. According to “www.murdermap.co.uk” the cases are updated regularly to show the progress of the investigation, (solved or unsolved case), however, the exact time interval of the updates is not stated. In addition we consider one more variable, i.e. the number of Borough’s offences per 1,000 of the population. The rates were retrieved from the Metropolitan Police Service [52] to examine whether the overall crime density of the Borough that includes the location of the incident may have an impact on the frequency of the tweets. In other words do crimes occurring in areas of high crime rates receive respectively higher public attention or vice versa?

**Fig 7:**
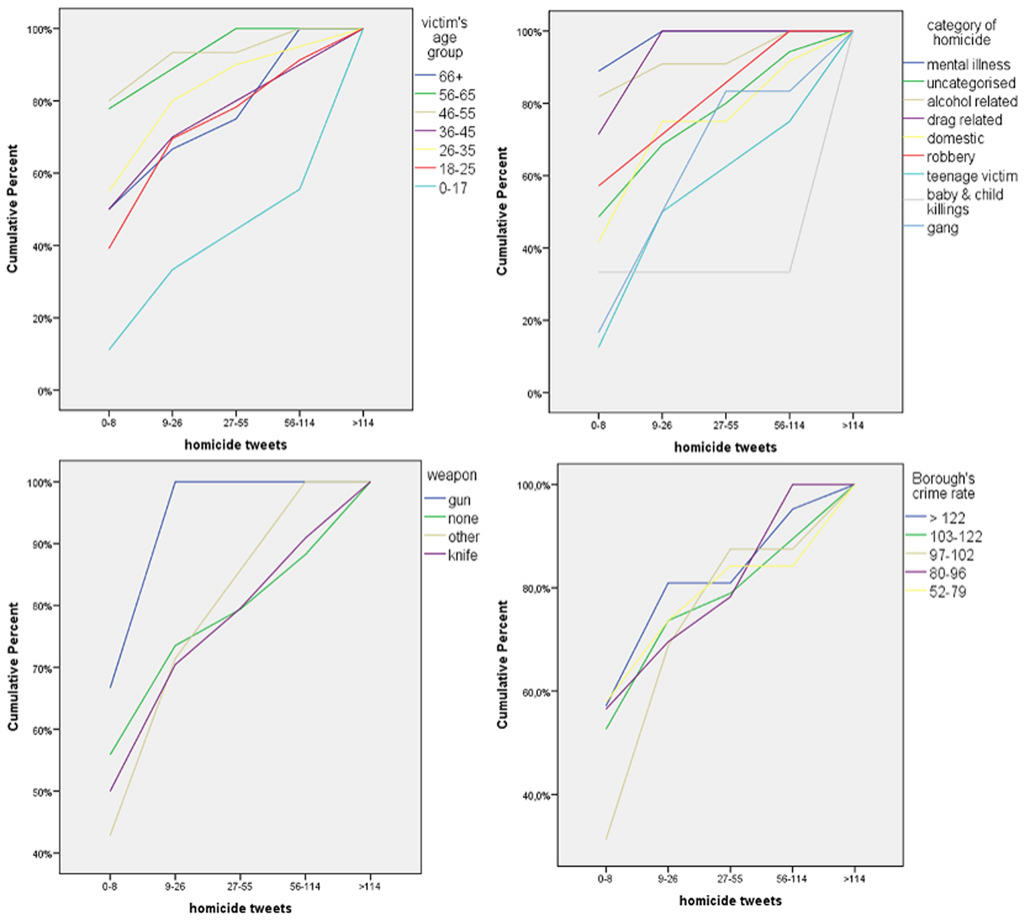
Cumulative percentages of the tweets’ frequency by categories of each variable.

The first step in developing the model was to observe the cumulative percentages of the tweets’ frequency by the categories of each variable. This was particularly useful for the non-binary categorical variables (more than two categories) since visual observations outlined which categories may act as predictors in the model and therefore should be selected as the categories which the others will be compared to. Fig. 7 illustrates four variables of the model (due to space constraints only four of the ten variables are illustrated). A general observation is that when a line is located more to the right side of the graph compared to the other lines then the respective category is associated with a higher tweets frequency. Hence, the victim’s age group “0–17” was selected to be compared with the remaining categories, since on the graph it greatly differs from the other age groups. For the category of homicide the “baby & child killings” and “teenage victims” categories were not selected because they correspond to a large degree with the victim’s age group “0–17” category. Therefore, the “gang” category (the next one with high tweets’ frequency) was selected. Regarding the weapon variable, both “knife” and “none” have similar trends, which are higher than the other two categories. The “knife” category was finally selected since it has more homicide tweets than the “none” category, in total. The last variable of Fig. 7, the Borough’s crime rate, does not have a clear trend in terms of its categories. In this case the highest class (>122) was selected to be compared with the others based on the assumption stated in the previous paragraph.

The model was developed using a stepwise procedure similar to a backward elimination of the variables with the largest p-value [53]. After a sequential removal of four of the ten variables (perpetrator’s gender, status of conviction, number of perpetrators, and victim’s gender) we obtained the optimal model. The model fit was significant and the Goodness-of-fit tests were insignificant (this means that the null hypothesis is true and the model is a good fit). Furthermore, the Pseudo R-square decreased insignificantly with the removal of each of the four variables (Negelkerke of original model with all ten variables: 0.738, Negelkerke of final model with six variables: 0.714). Finally, 71.4% of the variance of the model’s outcome is explained by the remaining six explanatory variables.

From the six variables of the final model the perpetrator’s nationality and the crime rate of the Borough were insignificant variables. The remaining four variables yielded significant results and are presented in Table 1. More specifically, for an additional victim being in any of the age groups: 18–25, 26–35, 46–55, 56–65, and 66+, controlling for the other variables in the equation, the odds of that homicide being in a higher tweets’ class are lower by a minimum of 99.7% compared to the age group of 0–17 years old. Similarly, for an additional homicide in the category of “teenage victim”, “drug related”, or “mental illness”, controlling for the other variables in the equation, the odds of that homicide being in a higher tweets’ class are lower by a minimum of 99.67% compared to the “gang” category. Also, for an additional homicide performed by a gun, controlling for the other variables in the equation, the odds of that homicide being in a higher tweets’ class are lower by 99.69% compared to the homicide being performed by a knife. Finally, for an additional victim being British, controlling for the other variables in the equation, the odds of that homicide being in a higher tweets’ class are more than 100 times higher than for a non-British victim.

**Table 1:** Parameter estimates of the OLR model (only significant variables are shown).

Explanatory variables		Estimate	Std. Error	Wald	Sig.	Exp(B)
**Age group**	66+	-14.538	4.235	11.781	.001	**0.000**
	56–65	-6.960	2.997	5.392	.020	**0.001**
	46–55	-9.324	2.827	10.874	.001	**0.000**
	36–45	-4.526	2.723	2.762	.097	
	26–35	-8.498	2.542	11.175	.001	**0.000**
	18–25	-5.822	2.099	7.694	.006	**0.003**
	***0–17***					
**Nationality**	victim is British	4.735	1.675	7.991	.005	**113.878**
	***victim is not British***					
**Category**	mental illness	-5.723	2.367	5.847	.016	**0.003**
	not categorised	-2.624	1.508	3.027	.082	
	alcohol related	-25.077	7.291.646	.000	.997	
	drug related	-6.802	2.426	7.861	.005	**0.001**
	domestic	.098	1.703	.003	.954	
	robbery	-2.464	1.938	1.617	.204	
	teenage victim	-5.784	2.229	6.730	.009	**0.003**
	baby & child killings	12.675	0.000			
	***gang***					
**Weapon**	gun	-5.784	2.219	6.793	.009	**0.003**
	none	.471	.870	.293	.588	
	other	2.717	1.798	2.284	.131	
	***knife***					

**Fig 8:**
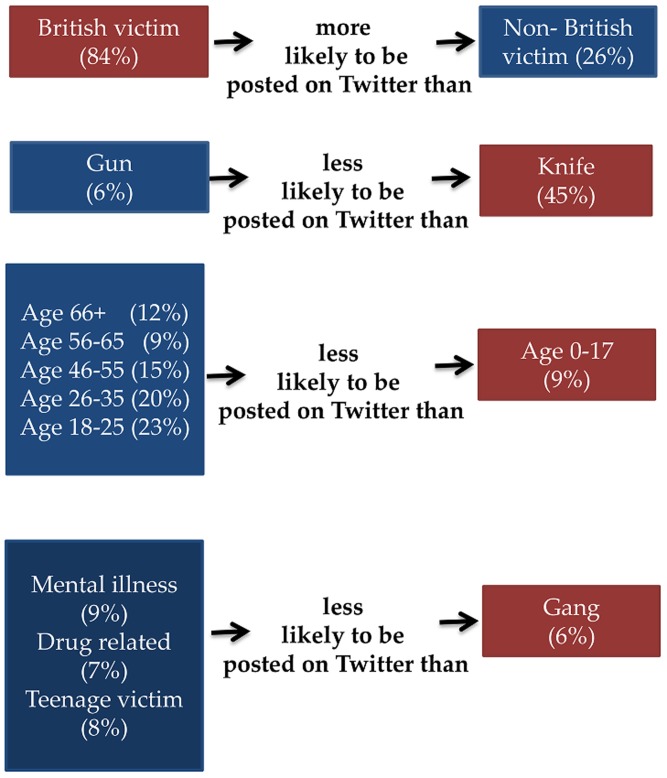
Posting likelihood on Twitter by category of the explanatory variable. Likelihood is expressed as odds, namely the likelihood of an event occurring to the likelihood of not occurring. Blue rectangles indicate categories of lower posting likelihood compared to the categories in red rectangles. The percentages in parenthesis show the frequency of this category in the *Homicide-Dataset*.

As a check of the regression homicide data, the significant variables as predictors of public concern are compared with the frequency of the categories in the *Homicide-Dataset* (Fig. 8). Nationality of a victim and weapon seem to be in line with their frequencies. For instance 84% of the victims were British and therefore it makes sense to expect more tweets regarding British victims. In the same manner, the weapon most commonly used in homicides was the knife, and respectively the likelihood of referring to such a crime is higher than posting about a homicide performed by a gun. On the other hand, the age and the crime’s category variables are not in accordance with their frequencies implying that certain topics receive high public concern even though they are not as frequent as others. In particular, homicides where the victim’s age is above 18, apart from the age group of 36–45, are less likely to be mentioned on Twitter, even though they are as frequent, or in most cases more frequent than the homicides where the victim was underage. Also, a “gang” homicide seems to trigger more concern than categories of higher occurrence such as “teenage victim”, “drug related”, and “mental illness”.

## Discussion

This study utilized Twitter data to explore the spatio-temporal dimensions of homicide incidents’ impact on the public, as well as the influence of the characteristics. To perform the analyses the data-processing methods `Links Correspondence Method' and ‘Home Estimation Method’ by Lampoltshammer et al. [41] were used. The validation of the proposed approach has been analyzed separately and can be found in the S1 Text. In short, the ‘Home Estimation Method’ was validated and proved to be an appropriate technique to estimate home locations of Twitter users. The aspects of crime concerns (i.e. temporal, spatial, and crime characteristics) were validated as well and the findings show that they are the main themes of discussion in homicide related tweets.

With regards to the temporal response of homicide news on Twitter, a clear temporal decay was observed with more than 2/3 of all tweets related to a particular homicide posted within one month of the incident. 81% of those were posted within a week and 26% on the day after the homicide. Furthermore, smaller tweeting peaks exist after five and seven months a crime has happened. This is likely explained by the release of new information about a case. Finally, additional analysis shows that temporal variations in crime-tweets frequencies are not related to the number of homicides in the respective months.

The spatial analysis revealed a strong spatial dependency between the estimated home locations of users who tweeted about homicide related news and the locations of these incidents. More precisely, the distance decay model of distances between these paired locations suggests that the number of messages regarding a particular homicide increases as the users’ locations become closer to the location of the homicide. This finding seems to be in line with findings of previous studies which confirmed that local crime news are associated with a higher level of fear of crime for local residents compared to non-local crime news [12], [13]. If people have a greater fear about homicides happening in their neighborhoods, then they are more likely to discuss them, also via Twitter posts, rather than addressing other crime news. It is also consistent with Tobler’s [54] first law of geography which states that *near things are more related than distant things*. People may be more affected by crime stories when they concern their familiar environment.

Analysis of crime characteristics indicates that some of them are associated with a higher frequency of tweets than others. In addition, previous studies used socio-demographic characteristics of people who are exposed to crime news as predictors of fear of crime. Here we used the characteristics of crime incidents as predictors of concern about crime and operationalized that concern via higher levels of posting frequency. The results showed that homicides that have at least one of these characteristics, namely being committed by a gang, involving an underage or a British victim, and using a knife as a weapon, are positively related with a higher frequency of tweets. Furthermore, homicides committed by a gang or underage victims were categories less numerous than others in the *Homicide-Dataset*. This finding implies that either the public is more sensitive to these types, or that the estimated crime prevalence for these types is higher than the actual crime prevalence. The latter explanation is consistent with findings from the literature regarding the crime rates perception gap [55–58].

On the other hand, other crime characteristics do not have a significant impact on the posting frequency. For instance, the crime rate of the Borough where the incident occurs does not have an impact on people’s decision on posting about it. This is consistent with findings from the fear of crime literature which suggest that personal risks do not consistently correspond with official statistics of neighborhoods [6]. However, the relationship that is examined here is rather specific. It shows that the background risk (crime rate of an area) does not affect human reactions to homicides. The results of the model may have been different if human reactions to other or all crime types were examined. For instance, tweets with general crime content may be strongly related with the underlying crime clustering in space. Also, tweets with general crime content may be somehow related with the underlying crime clustering with evidence of spatial variations. This relationship seems to be an unexplored opportunity that can be associated or assist current research efforts on crime prediction. Literature on predictive mapping utilizes past crime incidents and quantitative findings about repeat victimization to predict future incidents [59]. Crime related geo-referenced tweets could be an alternative data source for prediction. The advantage of social media data is that they can be collected and analyzed almost in real time. Thus, they may reveal trends and changes in the spatial distribution of crime related discussions that is important or correlated with future crime trends.

Legitimacy of Big data analytics and prediction has been already confirmed in many research areas, for instance “infodemiology” [60], [61] [23], stock market [62], consumer preferences [63], and global mobility patterns [29]. Through the practice of computational social science, digital traces that we leave behind have the potential to enhance our understanding of our lives and societies or even transform our current knowledge [64]. Our study and in particular the agreement between the received results and existing crime literature grounded in traditional survey approaches, proves that Twitter data may also be successfully applied for crime analysis. The spatial analytical approach that was used in this study and, in general, the examination of the spatial impact of events is perhaps only viable with user-generated georeferenced content. The practice of social sciences is moving into a new scientific era where human sensor data such as social media are not only effective and reliable for investigation, but also necessary to address key research questions.

## Supporting Information

S1 Dataset Aggregated anonymized data of the study. (XLSX)

S1 Text Validation of the approach. (DOCX)
