# Supplementary material for: Exploring Twitter to Analyze the Public’s Reaction Patterns to Recently Reported Homicides in London
Source: PLoS One. 2015 Mar 26;10(3):e0121848. doi: 10.1371/journal.pone.0121848 (PMC4374728; doi:10.1371/journal.pone.0121848)
Supplement: S1 Text — (DOCX) [file pone.0121848.s002.docx]

# Validation of the approach

This section provides a validation of the analytical parts of this study. The first subsection “Home Estimation Method” assesses the appropriateness of the method to estimate the home locations of the users. The second subsection “The aspects of crime concerns” employs a topic modeling method to demonstrate that the presented topics for analysis (**temporal, spatial, and frequency analysis**) have been reasonably selected.

# Home Estimation Method

We calculated the “first-order nearest neighbour index” (NNI) for the locations of each user **[1]**. The results of the tests revealed that the distributions of the users’ *night activity spaces* are extremely clustered. The indices range from 0 to 0.2238 and have a mean of 0.0323 at p= 0.0001. Also, the average “Mean Nearest Neighbour Distance” is 227.8241 meters. **Fig. 1** shows clusters of geo-located tweets of three users so as to give a visual aid on how *night activity spaces* may look like. In this figure, we selected users whose NNI is close to the minimum, the average, and the maximum values, so as to present different cases of geo-located tweets distributions. For privacy reasons the figure shows only the ellipses (clusters) of the users without any background information, which would allow to localize users’ activity spaces in the London area. The three users have different types of night activity spaces. For example night locations of User B are less dispersed than the other users (the maximum distance between cluster centroids is 5.9 km), though he or she has a more complicated travelling pattern (highest number of clusters) than the others. The length of the major axis of the highest frequency cluster ranges from 430 meters (User A) to 5,665 meters (User C), which translates to the size of a few blocks up to the size of a Borough. However, the most common pattern can be considered as that of User B, since it represents the average NNI. For User B, the majority of locations are distributed in a cluster of which the length of the major axis is 720 meters and it contains 76% of the locations. The spatial statistics’ results show that the majority of users mainly tweet from one place or a small area compared to their overall activity spaces.

**
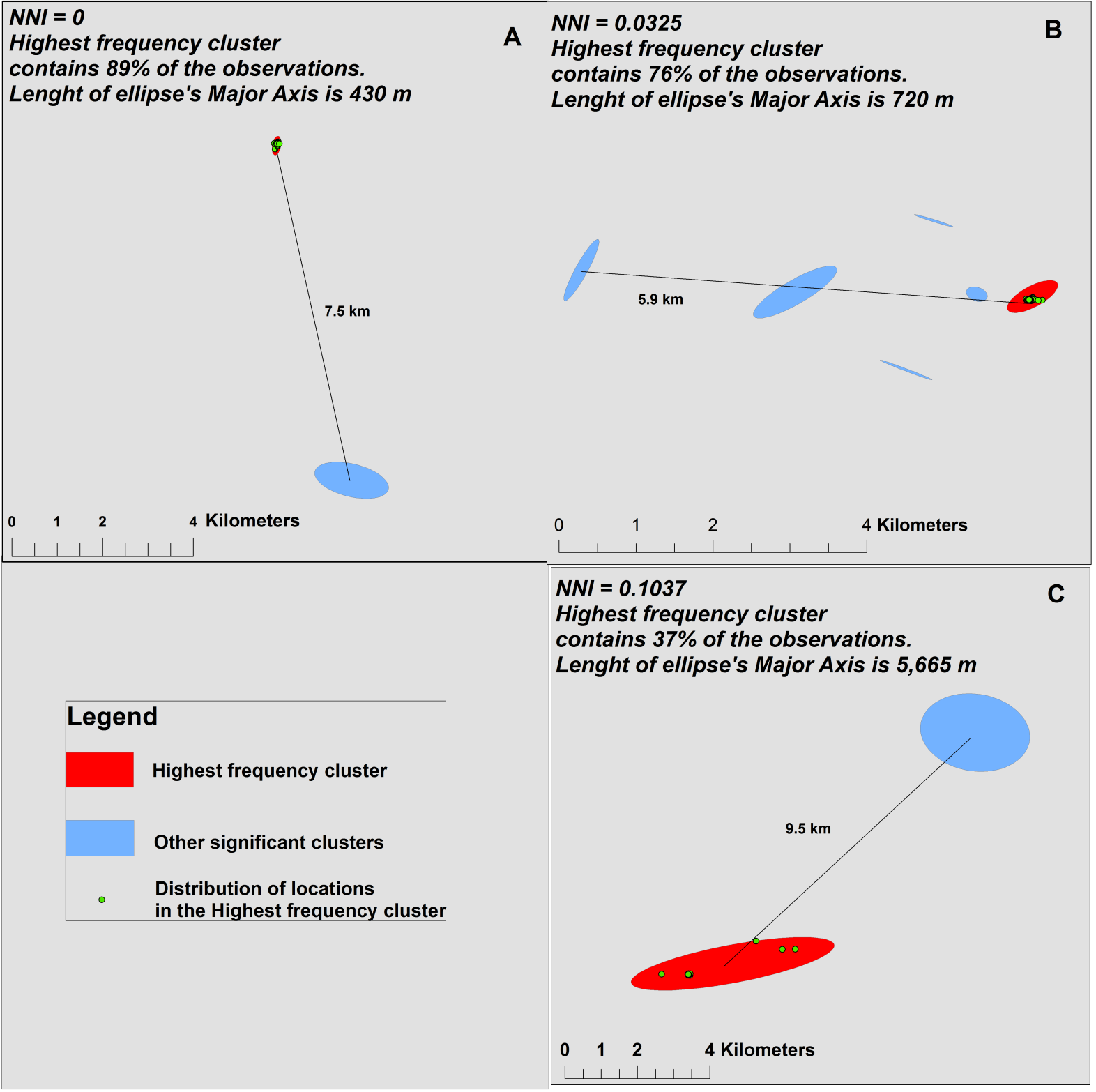
**

**Fig. 1. Significant clusters of geo-located tweets for users A, B and C.**

Furthermore, the validation spatial statistics (NNI and “Mean Nearest Neighbour Distance”) as well as the distance decay model (logarithmic function) presented in “**Spatial Analysis”** section were initially performed three times for the three subsets geo-located tweets: a) Users with more than 30 geo-located tweets, b) users with more than 20 geo-located tweets, and c) users with more than 10 geo-located tweets. First, the three subsets yielded similar analytical results. In addition to that, a Kruskal-Wallis test was performed on the distributions of the distances between the estimated home locations of users and the locations of homicides, which revealed that distributions are the same across the three subsets (H=0.6929, p=0.7072) **[2]**. Consequently, we propose that a threshold value of ten locations per user is adequate for performing this type of local analysis.

# The aspects of crime concerns

This study analyzed three aspects of crime concerns, namely the temporal, the spatial, and the crime characteristics. As an evaluation approach we want to see the degree of which these aspects are being represented, in terms of wording, within the corpus of the users messages (*HomicideTwitter-Dataset)*. To do this we used topic modeling, which according to Posner **[3]** gives meaningful improvements to simple word-frequency counts. Topic models are statistical language learning algorithms that cluster words of a corpus that are more likely to occur together. The clusters are called topics and according to Steyvers and Griffiths **[4]** are probability distributions over words. This approach offers an automated and accurate way to deal with word frequencies of a large body of unstructured text. The aim is to associate and compare prominent topics and words included in the *HomicideTwitter-Dataset* with the examined aspects that affect the likelihood of posting a homicide related tweet.

In particular, we used the topic modeling tool of the Mallet Toolkit [5]. This tool is java-based open source software, which performs the “Latent Dirichlet Allocation” (LDA) topic modeling for large collections of unlabeled text. The LDA algorithm is a three-level hierarchical Bayesian model developed by Blei et al. **[6]**. The Mallet topic modeling tool and its incorporated LDA algorithm have been extensively used in digital humanities research for exploring emerging topics from large volume documents such as blogging, newspapers, history, and archeology **[7]**. As a data source for our corpus we used a refined version of the HomicideTwitter-Dataset. Manual inspection of a tweets sample indicated that the majority of users were posting only the title and the URL of the media article (the URLs were the links of the look-up table). This can be explained by Twitter’s restrictions regarding the length of the text. Since we wanted to assess only the user’s opinion statements at this point, we only selected the tweets, which contained personal messages and from those we also deleted the article’s title and the URL. The new subset of personal messages contains 14% of the original dataset (455 tweets out of the 3,372). The topic modeling was performed to the refined subset and resulted in fifteen topics, described with fifteen words each. Each topic (word clusters) contains the most prominent topics (words). Number of topics was decided by multiple analyses, looking for robust results with a focus to allow nuance and uninterpretable topics to be detected. From the retrieved words all adjectives and adverbs that express emotions or opinions (e.g. awful, devastating, incredibly), as well as swear words were excluded. Consequently the remaining words (81% of the initial word total) consist of all nouns, verbs, and certain adjectives (e.g. young) that could potentially be related with the predictors.

Last, aspects to be inspected were separated into eight variables: 1) time, 2) space (user's location and Borough’s crime rate), category of homicide, weapon, victim’s age, nationality (perpetrator or victim), gender (perpetrator or victim), and the status of the case. The user's location and the Borough’s crime rate have been assigned together to the “space” category because if a topic includes a word such as the name of a neighborhood it is not clear whether the user mentions it because he or she lives there, or because this is an area with a high crime rate. Similarly, nationality and gender related words may refer to the perpetrator or the victim.

The matching of the topics’ words with the variables was subjectively performed by the authors. Results are presented in **Fig. 2** and show that the words’ meanings are so clear that it is unlikely that other individuals would perform a different matching with the variables. All variables have been identified within the fifteen most prominent topics of the users’ personal messages. This confirms the rationale of analyzing these variables. Also, **Table 1** shows in how many topics each variable was encountered (in total fifteen topics) and the percentage of such words in the total topics’ words. For instance, the word “English”, which was associated with the variable “nationality”, was encountered only once in one topic, thus this variable was found in 0.5 % of all words (one word out of the 182). The numbers of topics where words related to the variables were found ranges from 1 to 12 topics, and words related to space, gender, and age group were found in the vast majority of the topics. Lastly, 41% of the words (74 out of the 182) are related with the study’s predictors. Considering the amount of wording that can be used to express opinions, our result indicates that we selected an adequate set of variables that captures the main themes of discussion. In addition, the remaining words do not seem to address a particular topic that could be used as an additional variable to be analyzed.

**Table 1. Frequency of variables in the most discussed topics.**

Number of topics shows in how many topics a word related with the respective variable was encountered (in total 15 topics). Words’ percent shows the percentage of related words by variable to the total words of the topic modeling.

| **Variables** | **No. of topics** | **Words' percent** |
| --- | --- | --- |
| Time | 4 | 2.7% |
| Space | 11 | 8.2% |
| Category of homicide | 4 | 2.2% |
| Weapon | 6 | 3.8% |
| Age group | 8 | 5.5% |
| Nationality | 1 | 0.5% |
| Gender | 12 | 12.1% |
| Status of conviction | 8 | 5.5% |
|  | *Average = 6.8* | *Total= 41%* |


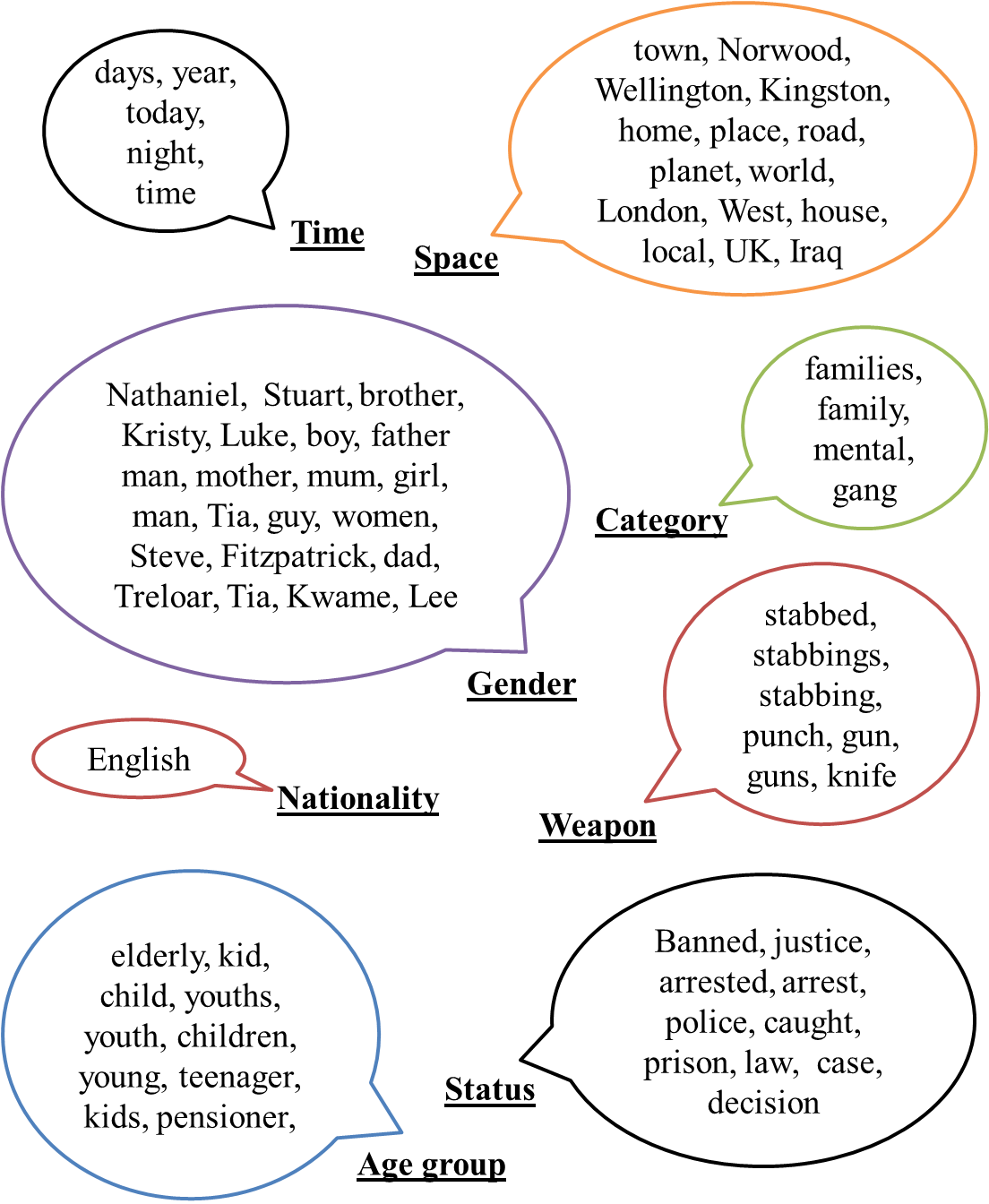


**Fig. 2. The topic modeling words paired with the study’s variables.**

# References

**1.** Clark, P. J., & Evans, F. C. (1954). Distance to nearest neighbor as a measure of spatial relationships in populations. Ecology, 445-453.

**2.** Kruskal, W. H., & Wallis, W. A. (1952). Use of ranks in one-criterion variance analysis. Journal of the American statistical Association, 47(260), 583-621.

**3.** Posner, M. Very basic strategies for interpreting results from the Topic Modeling Tool. Available: <http://miriamposner.com/blog/very-basic-strategies-for-interpreting-results-from-the-topic-modeling-tool/> .Accessed 29 May 2014.

**4.** Steyvers, M., & Griffiths, T. (2007). Probabilistic topic models. Handbook of latent semantic analysis, 427(7), 424-440.

**5.** McCallum, A. K. Mallet: A machine learning for language toolkit, 2002.

Available : <http://mallet.cs.umass.edu/> .Accessed 19 March 2014.

**6.** Blei, D. M., Ng, A. Y., & Jordan, M. I. (2003). Latent dirichlet allocation. the Journal of machine Learning research, 3, 993-1022.

**7.** Graham, S. & Milligan, I.. Review of MALLET, produced by Andrew Kachites McCallum, 2013. Available:

<http://journalofdigitalhumanities.org/2-1/review-mallet-by-ian-milligan-and-shawn-graham/> .Accessed 19 March 2014.
